# ﻿New records of the squat lobster genus *Munidopsis* Whiteaves, 1874 (Crustacea, Decapoda, Munidopsidae) from the deep sea off Taiwan

**DOI:** 10.3897/zookeys.1166.104009

**Published:** 2023-06-12

**Authors:** Masayuki Osawa, Tin-Yam Chan, Chien-Hui Yang

**Affiliations:** 1 Estuary Research Center, Shimane University, 1060 Nishikawatsu-cho, Matsue, Shimane 690-8504, Japan Shimane University Shimane Japan; 2 Institute of Marine Biology and Center of Excellence for the Oceans, National Taiwan Ocean University, 2 Pei-Ning Road, Keelung 202231, Taiwan National Taiwan Ocean University Keelung Taiwan

**Keywords:** COI, deep sea, distribution, Galatheoidea, new record, western Pacific

## Abstract

Two species of the squat lobster family Munidopsidae, *Munidopsisalbatrossae* Pequegnat & Pequegnat, 1973 and *M.pycnopoda* Baba, 2005, are reported from Taiwan for the first time based on specimens collected from lower bathyal depths. The Taiwanese material of *M.pycnopoda* also represents the first record of the species from the Pacific Ocean and greatly extends this species’ geographical range from the western Indian Ocean to western Pacific. The giant *Munidopsis* specimen from Taiwan is identified as *M.albatrossae* mainly by DNA barcoding even though *M.albatrossae* and *M.aries* (A. Milne-Edwards, 1880) are both morphologically and genetically extremely similar.

## ﻿Introduction

The galatheoid family Munidopsidae is recognized to include five extant genera (Ahyong et al. 2010, 2011; Rodríguez-Flores et al. 2023): *Galacantha* A. Milne-Edwards, 1880; *Janetgalathea* Baba & Wicksten, 1997; *Leiogalathea* Baba, 1969; *Munidopsis* Whiteaves, 1874; and *Shinkaia* Baba & Williams, 1998. Among these genera, *Munidopsis* is the most speciose and represented by about 300 species worldwide, although abyssal species might include fewer species than currently considered (cf. Rodríguez-Flores et al. 2022a, 2023). From Taiwanese waters, 37 species of the genus have been recorded (Baba et al. 2009; Lin and Chan 2011; Lin et al. 2013; Osawa et al. 2013), but recent deep-sea cruises have still discovered more species, including a giant *Munidopsis* specimen (carapace length including rostrum, 100.0 mm) never collected before. Close examination revealed that the giant specimen is *Munidopsisalbatrossae* Pequegnat & Pequegnat, 1973. Another newly recorded species is *M.pycnopoda* Baba, 2005, which was previously known only from the western Indian Ocean (Baba et al. 2008). The present paper reports these new findings and increases the total species number of *Munidopsis* in Taiwanese waters to 39.

## ﻿Materials and methods

The specimens examined are deposited in the National Taiwan Ocean University, Keelung (**NTOU**). Postorbital carapace length (pcl), an indication of specimen size, is measured in the dorso-midline from the level of the postorbital margin to the posterior margin of the carapace, and its width is measured between the lateral ends of the posterior cervical grooves, exclusive of spines or projections. Rostrum length is the dorsal distance between the anterior tip and the level of the postorbital margin, and rostrum width is between the left and right lateral bases of the ocular peduncles. Article lengths of chelipeds (P1) and ambulatory legs (P2–4) are measured along the dorsal and lateral midlines, exclusive of spines, respectively. Terminology used in the descriptions mainly follows that of Baba et al. (2009, 2011), except for the uses of “dorsal” and “ventral” for “extensor” and “flexor” margins, respectively, of the third maxilliped and ambulatory legs, and of “pleon” and “pleomere(s)” for abdomen and abdominal somite(s), respectively. Abbreviations used in the text include: Mxp3 (third maxilliped), P1 (first pereopod, cheliped), and P2–4 (second to fourth pereopods, first to third ambulatory legs). Photograph on fresh specimen was using the technique described by Chan et al. (2017).

For DNA barcoding sequence comparisons, crude genomic DNA was extracted from the muscle tissues of an ambulatory leg using QIAGEN DNeasy Blood and Tissue Kit (cat. no. 69504, Valencia, CA, USA) following the protocol of the manufacturer. Segments of mitochondrial COI were amplified by the universal primer set LCO1490/HCO2198 (Folmer et al. 1994). PCR reaction components, temperature cycling conditions and sequencing reaction followed those used by Ng et al. (2018). The assembled sequences were blasted (Basic Local Alignment Search Tool, BLAST, National Center for Biotechnology Information; NCBI) on GenBank for identifying the potential contamination and the closely related species.

## ﻿Taxonomic account

**Family Munidopsidae Ortmann, 1898**

### ﻿Genus *Munidopsis* Whiteaves, 1874

#### ﻿ Munidopsis albatrossae

Taxon classificationAnimaliaDecapodaMunidopsidae

Pequegnat & Pequegnat, 1973

F6D6BC03-9943-568B-88E5-C6341DE0867B

Figs 1 , 2

Munidopsis sp.—Wolff, 1961: 148, fig. 16.
Munidopsis albatrossae Pequegnat & Pequegnat, 1973: 163, figs 1, 2 (type locality: south of Madalena Bay, Baja California, 23°23.5'N, 112°30'W, 3219 m)—Hendrickx and Harvey 1999: 376 (list). Stevens 2002: 6 (list). Baba 2005: 280 (key), 284 (key, synonymies). Jones and Macpherson 2007: 480, fig. 2A. Baba et al. 2008: 131 (compilation). García Raso et al. 2008: 1282, fig. 2. Dong et al. 2019: 5, figs 1–3A.
Munidopsis aries —Ambler, 1980: 17. Wicksten 1989: 315 (list) [not M.aries (A. Milne-Edwards, 1880)].
? Munidopsisalbatrossae—Arnés-Urgellés et al. 2020: 18, fig. 12. Rodríguez-Flores and Schnabel 2023: 6, table S1.

##### Material examined.

Taiwan: 1♀ parasitized by rhizocephalan (pcl 77.8 mm), east off Taiwan, station CP4216, 24°15.02'–24°09.46'N, 122°11.55'–122°10.37'E, 2360–2928 m, French beam trawl, 16 Jan. 2021 (NTOU A01452).

**Figure 1.**
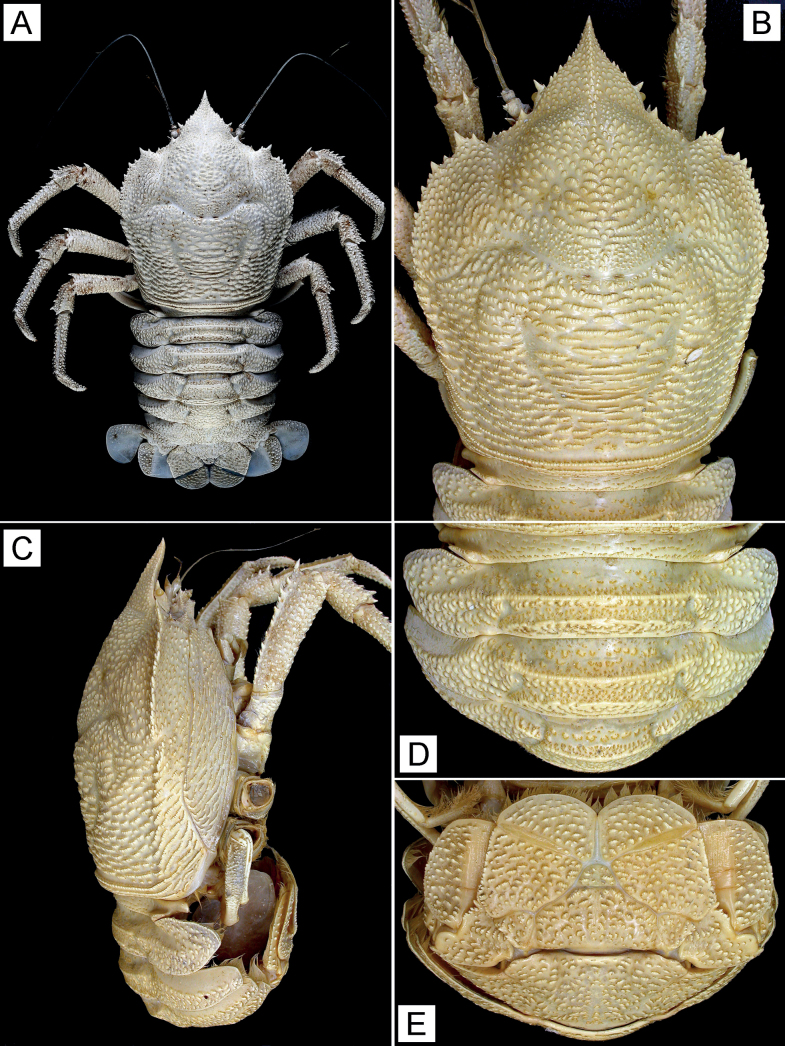
*Munidopsisalbatrossae* Pequegnat & Pequegnat, 1973, female parasitized by rhizocephalan (pcl 77.8 mm), east off Taiwan, station CP4216, NTOU A01452 **A** entire animal, dorsal view (lacking both P1) **B** carapace, dorsal view **C** carapace and pleon, right lateral view **D** pleomeres 1–4, dorsal view **E** pleomere 6 and telson, external view. Fresh coloration (**A**), preserved coloration (**B–E**).

##### Diagnosis.

Carapace (excluding rostrum) approximately as long as wide; dorsal surface with numerous transverse ridges and pair of small epigastric processes; front margin oblique; outer orbital angle with short spine (antennal spine); lateral margin decreasing in width posteriorly; anterior branchial margin convex, crested, with row of stout spines generally decreasing in size posteriorly. Rostrum 0.3 length of remaining carapace, slightly shorter than basal width, triangular, strongly narrowed on anterior half; dorsal surface with median carina. Sternite 3 subquadrate in general outline; sternite 4 narrowed and elongated anteriorly, anterior margin narrower than posterior margin of sternite 3. Pleon entirely unarmed; tergites 2 and 3 each with 2 elevated, blunt transverse ridges and many small ridges; posteromedian lobe of tergite 6 almost transverse, straight; telson composed of 8 plates. Ocular peduncle short, somewhat movable dorsoventrally, with strong mesiodorsal eye-spine directed anterolaterally. Article 1 of antennular peduncle with distolateral and distodorsal spines, distolateral spine larger. Antennal peduncle nearly reaching tip of mesiodorsal eye-spine by full length of article 4; article 1 with distomesial spine reaching midlength of article 2; article 2 with distolateral spine reaching midlength of article 3. Mxp3 merus with 2 or 3 small teeth on ventral margin, dorsal margin with small distal spine. P2–4 comparatively short; meri each with row of irregular sized spines on dorsal margin, ventrolateral margins slightly crenulated by short ridges; propodi gently narrowing from proximal to distal, each with dorsolateral and dorsomesial rows of small spines; dactyli approximately two-thirds length of propodi, ventral margins nearly straight, each with 8 or 9 teeth. Epipods absent from P1–4.

**Figure 2.**
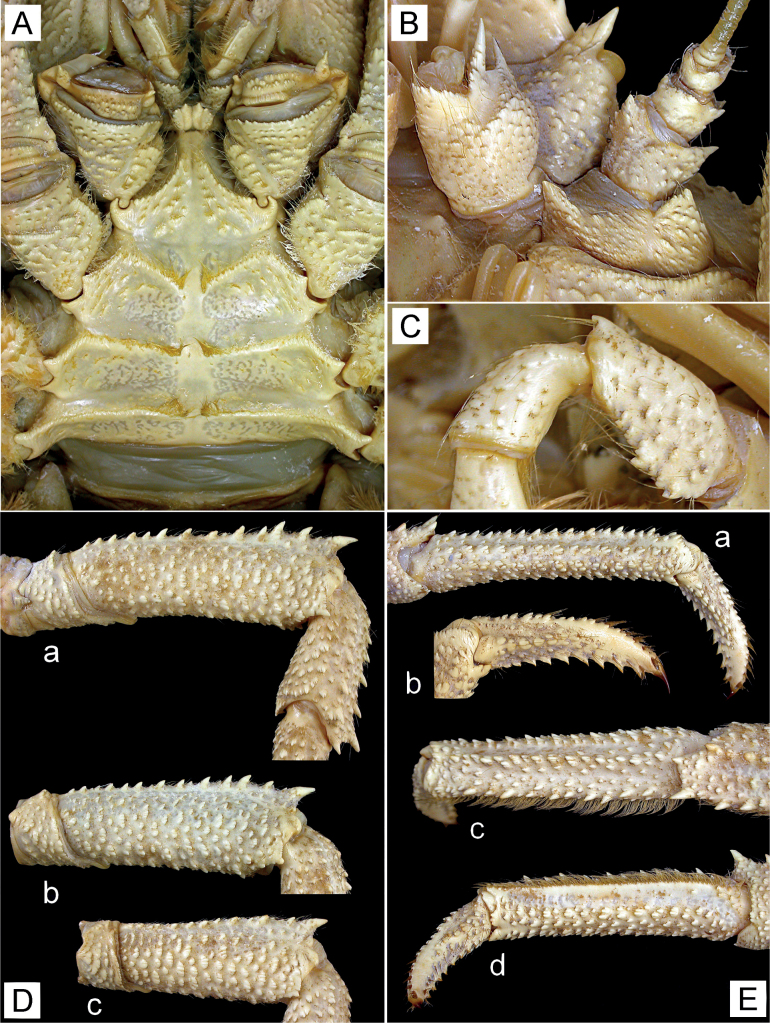
*Munidopsisalbatrossae* Pequegnat & Pequegnat, 1973, female parasitized by rhizocephalan (pcl 77.8 mm), east off Taiwan, station CP4216, NTOU A01452 **A** sternal plastron, ventral view **B** left eye, article 1 of antennular peduncle, and antennal peduncle, ventral view **C** left Mxp3, merus and carpus, lateral view **D** left P2–4, meri, lateral view (**a** P2, **b** P3, **c** P4) **E** left P3 (**a** propodus and dactylus, lateral view; **b** dactylus, lateral view; **c** propodus, dorsal view; **d** propodus and dactylus, mesial view).

##### Description.

Ridges on carapace and P2–4 each bearing row of minute granules and very short plumose setae. P2–4 with row of dense, short, plumose setae on dorsomesial margin of each propodus; coarse, short stiff setae on surfaces of each dactylus.

***Carapace*** (Fig. 1A–C) excluding rostrum approximately as long as wide. Dorsal surface moderately arched from side to side; anterior half with numerous, short arcuate ridges; posterior half with longer transverse ridges, posteriormost ridge uninterrupted, somewhat elevated, preceded by shallow groove; pair of epigastric processes small, weakly raised; anterior cervical groove shallow, posterior cervical groove deep. Front margin oblique. Orbit slightly excavated, outer orbital angle with short but prominent spine (antennal spine). Lateral margin decreasing in width posteriorly; anterior branchial margin convex, crested, with row of stout spines generally decreasing in size posteriorly, anterior second spine strongest; anterolateral spine smaller than outer orbital spine, located below level of anterior branchial margin; posterior branchial margin nearly straight, dorsally with shallow, broad sulcus. Rostrum (Fig. 1A–C) approximately 0.3 length of remaining carapace, slightly shorter than basal width, triangular, slightly directed ventrally; anterior half strongly narrowed, with acute tip; dorsal surface with median carina not extending to epigastric region; lateral margin ridged, with short stout spine medially; ventral surface flattish, with blunt median carina.

***Sternal plastron*** (Fig. 2A) slightly wider than long. Sternite 3 subquadrate; anterior margin with small denticles, subdivided into 2 lobes by shallow median notch, each lobe with shallow longitudinal groove; small protuberance present below median notch. Sternite 4 narrowed and elongated anteriorly; anterior margin narrower than posterior margin of sternite 3; lateral margins each with row of small tubercles on posterior half; ventral surface slightly concave, with median groove anteriorly and scattered short ridges posteriorly.

***Pleon*** (Fig. 1A, C–E) entirely unarmed, with numerous flattened tubercles on lateral parts of pleomeres 2–4. Tergite 1 with sparse, short transverse ridges. Tergites 2 and 3 each with 2 elevated, blunt transverse ridges; entire regions bearing many small ridges. Tergite 4 with elevated, blunt transverse ridge anteriorly. Tergites 5 and 6 each with obsolete ridge anteriorly on each side; surfaces covered with short ridges; posteromedian lobe of tergite 6 almost transverse, straight, lateral lobes weakly produced. Telson (Fig. 1E) distinctly wider than long, composed of 8 plates being numerous, short ridges.

***Ocular peduncles*** (Figs 1A–C, 2B) short, somewhat movable dorsoventrally, with strong mesiodorsal eye-spine directed anterolaterally. Cornea small, approximately equal to basal width of mesiodorsal eye-spine, not pigmented.

***Article 1 of antennular peduncle*** (Fig. 2B) with distolateral and distodorsal spines, distolateral spine larger; ventrodistal margin with row of small denticles; dorsomesial distal angle with minute spine; ventral surface with small, flattened tubercles on convex lateral part.

***Antennal peduncle*** (Figs 1B, 2B) nearly reaching tip of mesiodorsal eye-spine by full length of article 4. Article 1 with distomesial spine reaching midlength of article 2; distolateral spine small, blunt. Article 2 with small distomesial spine ventrally, distolateral spine reaching midlength of article 3; surfaces with sparse, small denticles and tubercles. Article 3 with small distomesial spine dorsally, distal margin with row of small denticles. Article 4 with minute denticles on distal margin.

***Mxp3 merus*** (Fig. 2C) with 2 or 3 small teeth on proximal half of ventral margin; dorsal margin with small distal spine; carpus unarmed.

***P1*** missing.

***P2–4*** (Figs 1A, C, 2D, E) comparatively short, somewhat compressed laterally. Meri decreasing in size posteriorly, P3 0.9 length of P2, P4 0.8 length of P3; dorsal margins bluntly crested, each with row of irregularly sized spines, terminal spine strongest; lateral surfaces covered with short, transverse or arcuate ridges; ventrolateral margins rounded, terminal angle with small spine (P2) and small tubercle (P3 and P4). Carpi each with 3–5 distinct spines and some much smaller proximal spines on dorsal crest, terminal spine strongest; lateral surfaces with sparse, small, arcuate or transverse ridges entirely and each with somewhat elevated, longitudinal ridge of small spines (P2) and small protuberances (P4) on midline. Propodi approximately 5.0 times length of proximal height, gently narrowing from proximal to distal; dorsal surface flattish, dorsolateral and dorsomesial margins each with irregular row of small spines, dorsomesial spines stronger; lateral surfaces each with row of short, somewhat elevated ridges and blunt spines near ventrolateral margin; ventrolateral margins also crenulated by short, somewhat elevated ridges and blunt spines; ventral surfaces with numerous, small, arcuate ridges; distoventral margins each with 2 rounded, tuberculate lobes bearing 0 or 1 minute corneous spine. Dactyli approximately two-thirds length of propodi, moderately slender; surfaces with small, somewhat elevated ridges, dorsal surface flattish, lateral and mesial surfaces convex on each proximal midline; ventral margins nearly straight, each with 8 or 9 proximally diminishing teeth, each tooth terminating in acute corneous spine; terminal claws short, curved.

***Epipods*** absent from P1–4.

##### Coloration.

Body and appendages entirely white (Fig. 1A).

##### Distribution.

Eastern Pacific: Alaska Bay, off Oregon, Monterey Bay, Baja California, East Pacific Rise and west of Costa Rica; 2550–3680 m depths (Stevens 2002; Baba et al. 2008). Western Pacific: Weijia Guyot (12°43.01'N, 156°27.20'E) and Hawaii, 3225 m depth (Dong et al. 2019). Antarctic waters: Bellingshausen Sea, 1920 m depth (García Raso et al. 2008). In addition, material identified as *M.albatrossae* based solely on ROV images have been reported from the Galapagos Islands, 3392 m depth, and Hawaii Archipelago, between 3250 m and 3255 m (Arnés-Urgellés et al. 2020; Rodríguez-Flores and Schnabel 2023). The present specimen collected from off Taiwan at the depths of 2360–2928 m represents the second confirmed record of *M.albatrossae* from the western Pacific (Fig. 5).

##### Remarks.

The present large specimen lacks both chelipeds and was parasitized by an unidentified rhizocephalan on the internal surface of the pleon.

As discussed by previous authors (Pequegnat and Pequegnat 1973; Gore 1983; Jones and Macpherson 2007; García Raso et al. 2008), *M.albatrossae* and *M.aries* (A. Milne-Edwards, 1880) are morphologically very similar. Recorded distributions show that *M.albatrossae* occurs only in the Pacific including the eastern Pacific side of the Antarctic (Fig. 5), whereas *M.aries* ranges from the North Atlantic to the South Atlantic and also occurs off Reunion Island in the southwestern Indian Ocean (Macpherson and Segonzac 2005; Macpherson 2007).

Pequegnat and Pequegnat (1973) argued that *M.albatrossae* differed from *M.aries* (as *M.sundi* Sivertsen & Holthuis, 1956) by the following characters: (1) setose ridges on carapace more pronounced than in *M.aries*; (2) anterior branch of cervical groove not as distinct; (3) frontal margin of carapace noticeably less broad; (4) first spine behind anterior branch of cervical groove not appreciably larger than other lateral spines, considerably larger in *M.aries*; (5) posterior margin of carapace relatively broader than in *M.aries*; (6) pleomere 1 not completely smooth, but with four rows of weak, interrupted ridges and setose grooves on posterior portion; (7) anterior halves of pleomeres 2–4 not all smooth as in *M.aries*, but bearing interrupted setose ridges; (8) eyes more freely movable than in *M.aries*. However, these distinguishing characters except for the surface structures on the pleomeres 2–4 vary and are not so evident in the published specimens referred to *M.albatrossae* and *M.aries* (or as *M.sundi*). The present specimen from off Taiwan agrees more closely with *M.albatrossae* in the dorsal surfaces of the pleomeres 2–4 with many interrupted, short ridges (Fig. 1D), unlike being apparently smooth and no such ridges in *M.aries* (cf. Sivertsen and Holthuis 1956: pl. 4 fig. 2, as *M.sundi*; Macpherson and Segonzac 2005: fig. 3). The development of the carapace epigastric process varies in previous accounts of *M.albatrossae*; the processes are illustrated as rather distinct (Pequegnat and Pequegnat 1973; García Raso et al. 2008; Dong et al. 2019) or indistinct (Jones and Macpherson 2007) as in the present specimen (Fig. 1A–C).

A comparison of the barcoding segments of the COI gene (657 bp) of the Taiwanese specimen (GenBank accession number OQ996536) and *M.albatrossae* materials from the eastern Pacific (GenBank accession number DQ677692; Monterey Bay) and western Pacific (GenBank accession number MN397920; Weijia Guyot) reported by Jones and Macpherson (2007) and Dong et al. (2019) revealed only 0.19% and 0.35% sequence divergence, respectively. Meanwhile, there is 1.75% COI sequence divergence between the Taiwanese specimen and the northeastern Atlantic material reported as *M.aries* (GenBank accession number DQ677691; off Guinea) by Jones and Macpherson (2007; more details on this specimen, see Macpherson and Segonzac 2005).

The mitochondrial COI gene is considered as rather conservative in the genus *Munidopsis* between populations and among sibling species (cf. Jones and Macpherson 2007; Thaler et al. 2014; Coykendall et al. 2017; Dong et al. 2019; Rodríguez-Flores et al. 2023). Thus, the phylogenetic hypothesis based on COI data by Rodríguez-Flores et al. (2023) concluded that *M.albatrossae* and *M.aries*, as well as many other abyssal species of this genus, are not distinguished as different species. Nevertheless, such low genetic divergences may be more apparent than real because increasing depth could reduce the rate of substitution (Rodríguez-Flores et al. 2022b). While the number of specimens and genetic data required to determine the exact taxonomic status of these abyssal *Munidopsis* species are still very limited, for the time being we regard *M.albatrossae* and *M.aries* as separate species. The present Taiwanese specimen is hence assigned to *M.albatrossae* for having a higher genetic similarity and closer morphological matches (i.e., surface structure of pleomeres) to this species.

#### ﻿ Munidopsis pycnopoda

Taxon classificationAnimaliaDecapodaMunidopsidae

Baba, 2005

4297A649-660E-5E48-895A-BAA0CB41A061

Figs 3 , 4

Munidopsis pycnopoda Baba, 2005: 176, fig. 84 (type locality: Mozambique Channel, 14°20'S, 45°09'E, 3485 m), 279 (key), 293 (synonymy)—Macpherson 2007: 96. Baba et al. 2008: 156 (complement).

##### Material examined.

Taiwan: 2 ovigerous ♀♀ (pcl 12.2, 12.3 mm), east off Taiwan, station CP5292, 23°58.16'–23°55.25'N, 122°22.39'–122°21.60'E, 3575–3590 m, French beam trawl, 10 Aug. 2022 (NTOU A01453).

##### Diagnosis.

Carapace (excluding rostrum) slightly longer than wide; dorsal surface with numerous transverse ridges and pair of distinct epigastric spines; front margin oblique; outer orbital angle with prominent spine (antennal spine); lateral margin slightly convex, with 7 spines, first anterolateral, second to fifth spines and sixth and seventh spines on anterior and branchial margins, respectively, second spine strongest. Rostrum 0.4 length of remaining carapace, slightly shorter than basal width, narrowly triangular; dorsal surface with median carina. Sternite 3 subquadrate in general outline; sternite 4 narrowed and elongated anteriorly, anterior margin narrower than posterior margin of sternite 3. Pleon entirely unarmed; tergites 2 and 3 each with 2 elevated, blunt transverse ridges; posteromedian lobe of tergite 6 nearly transverse, straight; telson composed of 8 plates. Ocular peduncle short, faintly movable dorsoventrally, with 2 distinct eye-spines, mesiodorsal spine stronger than lateral spine. Article 1 of antennular peduncle with distolateral and distodorsal spines, distolateral spine larger. Antennal peduncle overreaching tip of mesiodorsal eye-spine by full length of article 4; article 1 with distomesial spine reaching midlength of article 2; article 2 with distolateral spine reaching midlength of article 3. Mxp3 merus with 5–7 small teeth on ventral margin, dorsal margin with small distal spine. P1 short, approximately as long as postorbital carapace, covered with short ridges on surfaces; chela 2.1 times as long as wide, without spines; fixed finger with short denticulate carina on distolateral margin; dactylus slightly shorter than palm, distinctly narrower than fixed finger. P2–4 moderately long, P2 overreaching tip of P1 by full length of P2 dactylus; meri each with row of distinct, irregular sized spines on dorsal margin, ventrolateral margins each with row of small spines and somewhat elevated, short ridges; propodi nearly equal in height from proximal to distal, dorsolateral and dorsomesial margins each crenulated by row of short ridges, dorsomesial margin usually with 1 or 2 distinct spines on proximal half; dactyli 0.5–0.6 length of propodi, ventral margins nearly straight, each with 12–14 teeth. Epipods present on P1, absent from P2–4.

##### Description.

Ridges on carapace and P1–4 each bearing row of minute granules and very short plumose setae. Coarse, short to moderately long setae on surfaces of P1–4.

**Figure 3.**
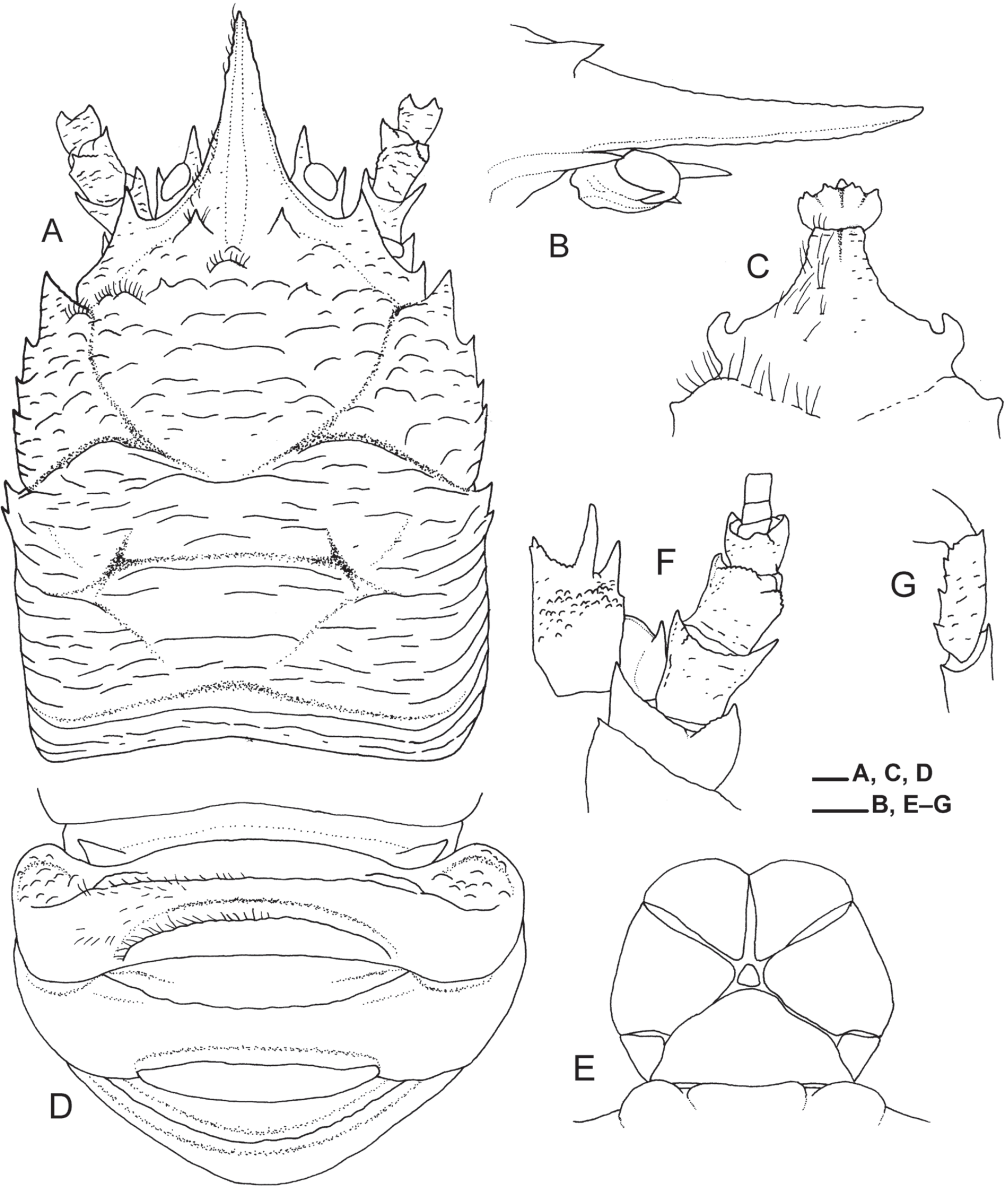
*Munidopsispycnopoda* Baba, 2005, ovigerous female (pcl 12.3 mm), east off Taiwan, station CP5292, NTOU A01453 **A** carapace, eyes, and antennal peduncles, dorsal view (setae partially drawn on left side) **B** rostrum and eye, right lateral view **C** anterior part of sternal plastron, ventral view (setae drawn only on right side) **D** pleomeres 1–4, dorsal view (setae drawn only on left side of pleomere 2) **E** telson and posterior margin of pleomere 6, external view (setae not drawn) **F** left eye, article 1 of antennular peduncle, and antennal peduncle, ventral view (setae not drawn) **G** left Mxp3, merus, lateral view (setae not drawn). Scale bars: 1.0 mm.

***Carapace*** (Fig. 3A) excluding rostrum slightly longer than wide. Dorsal surface moderately arched from side to side, with pair of epigastric spines; anterior half with arcuate ridges; posterior half with longer transverse ridges, posteriormost ridge uninterrupted, elevated, preceded by shallow groove; cervical grooves distinct. Front margin oblique, slightly concave behind ocular peduncle. Orbit slightly excavated, outer orbital angle with prominent spine (antennal spine). Lateral margin slightly convex, with 7 spines; first spine anterolateral, small (abnormally absent on left side in ovigerous female, pcl 12.3 mm); second to fifth spines and sixth and seventh spines on anterior and branchial margins, respectively, second spine strongest, directly behind end of anterior cervical groove. Rostrum (Fig. 3A, B) 0.4 length of remaining carapace, slightly shorter than basal width, narrowly triangular, horizontal or slightly upcurved distally; dorsal surface with median carina extending to epigastric region; lateral margin ridged, minutely crenulated distally; ventral surface flattish.

***Sternal plastron*** (Fig. 3C) slightly wider than long. Sternite 3 subquadrate; anterior margin with small denticles, subdivided into 2 lobes by shallow median notch; small protuberance present below median notch. Sternite 4 narrowed and elongated anteriorly; anterior margin narrower than posterior margin of sternite 3; ventral surface concave, with median groove anteriorly.

***Pleon*** (Fig. 3D, E) entirely unarmed. Tergites 2 and 3 each with 2 blunt transverse ridges, anterior ridge strongly elevated, posterior ridge preceded by shallow groove. Tergite 4 with blunt transverse ridge anteriorly and obsolete ridge posteriorly on each side. Tergites 5 and 6 each with obsolete ridge anteriorly on each side; posteromedian lobe of tergite 6 nearly transverse, straight, lateral lobes weakly produced. Telson (Fig. 3E) distinctly wider than long, composed of 8 plates.

***Ocular peduncles*** (Fig. 3A, B, F) short, faintly movable dorsoventrally; 2 or 3 eye-spines present, mesiodorsal spine strong and directed forward, lateral spine small, mesioventral spine tiny or absent. Cornea relatively narrow, maximum width distinctly less than width of rostrum at midlength, not pigmented.

***Article 1 of antennular peduncle*** (Fig. 3F) with distolateral and distodorsal spines, distolateral spine larger; ventrodistal margin with small spine mesially; ventral surface with small denticles on anterior half.

***Antennal peduncle*** (Fig. 3A, F) overreaching tip of mesiodorsal eye-spine by full length of article 4. Article 1 with distomesial spine reaching midlength of article 2, distolateral spine small. Article 2 with small distomesial spine ventrally, distolateral spine reaching midlength of article 3. Article 3 with small distomesial spine dorsally, distolateral margin granular. Article 4 unarmed.

***Mxp3 merus*** (Fig. 3G) with 5–7 small teeth on ventral margin, proximal 1 or 2 teeth stronger; dorsal margin with small distal spine; carpus unarmed.

**Figure 4.**
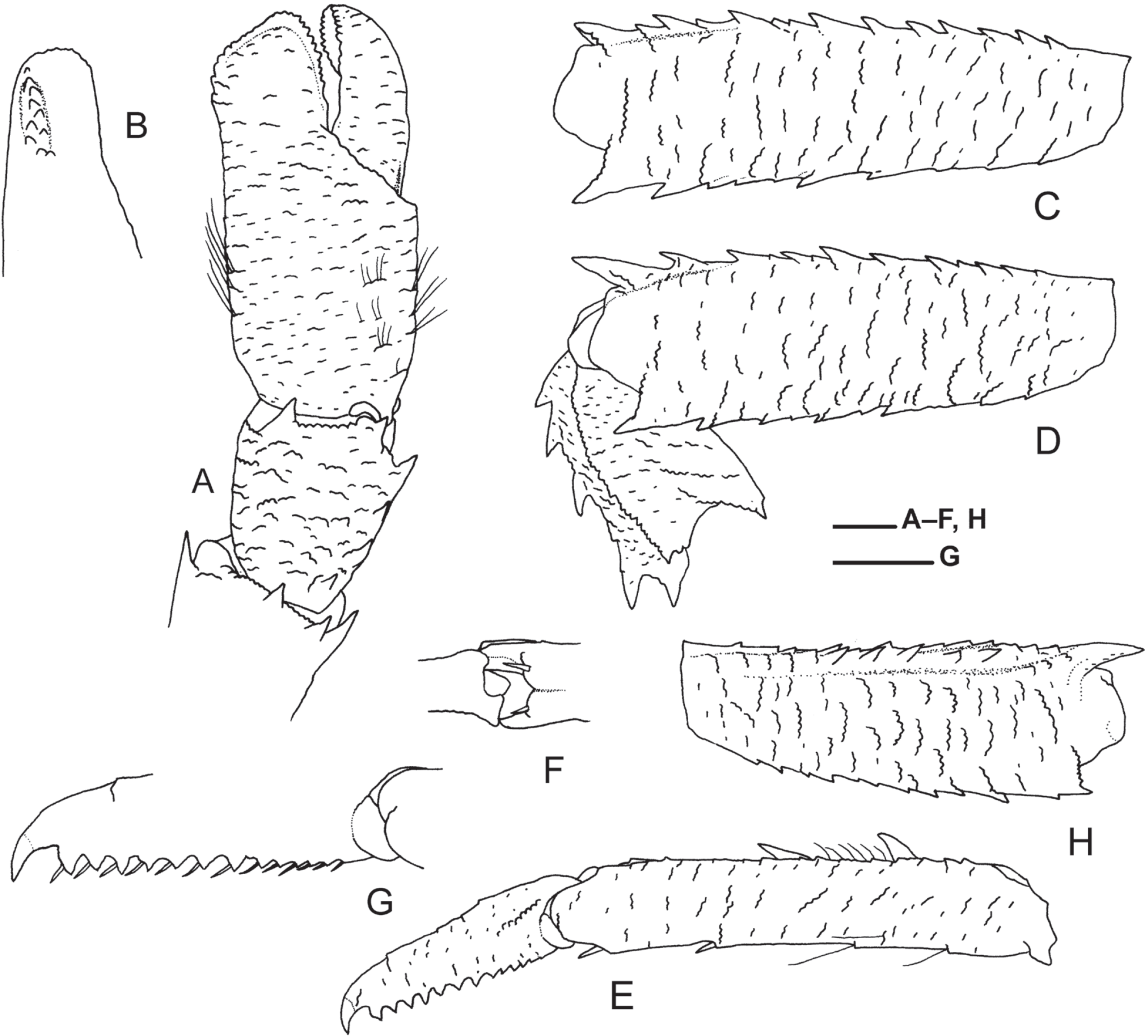
*Munidopsispycnopoda* Baba, 2005, ovigerous female (pcl 12.3 mm), east off Taiwan, station CP5292, NTOU A01453 **A** left P1, carpus and chela, dorsal view (setae partially drawn on palm) **B** left P1, fixed finger, lateral view **C** left P2, merus, lateral view (setae not drawn) **D** left P3, merus and carpus, lateral view (setae not drawn) **E** left P3, propodus and dactylus, lateral view (setae partially drawn) **F** left P3, distal part of propodus, ventral view **G** left P3, ventral margin of dactylus, lateral view **H** right P4, merus, lateral view (setae not drawn). Scale bars: 1.0 mm.

***P1*** (Fig. 4A, B) short, approximately as long as postorbital carapace, overreaching tip of rostrum by almost full length of chela, covered with short, transverse or scaly ridges on surfaces. Merus reaching nearly midlength of rostrum, with 5 distal spines (3 dorsal, 1 ventromesial, 1 ventrolateral); dorsal surface with irregular row of small spines on midline. Carpus nearly as long as wide, dorsal surface flattish, dorsodistal margin with 1 or 2 spines, mesial spine reduced in size or replaced by blunt tubercle; dorsomesial margin with row of 3 or 4 spines, distal most spine largest; laterodistal margin with small spine or unarmed; ventral surface smooth. Chela 2.1 times as long as wide. Palm slightly longer than wide, unarmed; dorsal surface somewhat convex from side to side. Fingers distally spooned; occlusal margins each distally with row of small denticles; fixed finger with short denticulate carina on somewhat inflated distolateral margin. Dactylus slightly shorter than palm, distinctly narrower than fixed finger.

***P2–4*** (Fig. 4C–H) moderately long, somewhat compressed laterally; P2 overreaching tip of P1 by full length of P2 dactylus. Meri decreasing in size posteriorly, P3 0.9 length of P2, P4 0.8 length of P3; dorsal margins bluntly crested, each with row of irregularly sized spines, terminal spine on each P3 and P4 strongest; lateral surfaces with short, transverse or arcuate ridges; ventrolateral margins each with row of small spines and somewhat elevated, short ridges, terminal spine on each P2 and P3 strongest. Carpi each with 4 or 5 spines on dorsal crest; lateral surfaces each with elevated, longitudinal ridge subparalleling dorsal crest. Propodi about 5.6 times as long as high, nearly equal in height from proximal to distal; dorsal surface flattish, dorsolateral margin crenulated by row of short ridges, dorsomesial margin also crenulated and with 1 or 2 (P2 and P3) and 0 or 1 (P4) distinct spines on proximal half; lateral surfaces with sparse, short ridges; ventral surfaces with 0 or 1 corneous spine on distal one-third; distoventral margins each with 2 corneous spines. Dactyli 0.5–0.6 length of propodi, moderately slender; ventral margins nearly straight, each with 12–14 proximally diminishing teeth, each tooth bearing short bristle-like setae; terminal claws short, curved.

***Epipods*** present on P1, absent from P2–4.

##### Coloration.

Body and appendages entirely white.

##### Distribution.

**Figure 5.**
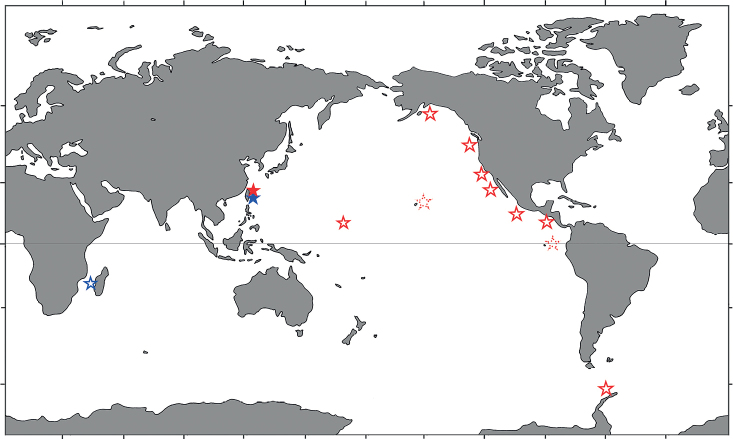
Global geographic distributions of *Munidopsisalbatrossae* Pequegnat & Pequegnat, 1973 (red) and *M.pycnopoda* Baba, 2005 (blue). Dotted line symbols are records based only on ROV images. Shaded symbols refer to new records in this work.

Previously only known from the Mozambique Channel at depths of 3450–3485 m (Baba et al. 2008). The present material collected from off Taiwan at depths of 3575–3590 m represents the first record *M.pycnopoda* from the Pacific Ocean and greatly extends the geographic range of this species (Fig. 5).

##### Remarks.

Baba (2005) originally described *M.pycnopoda* based on three specimens from the Mozambique Channel, with a note of intraspecific variation. The two specimens examined from off Taiwan agree well with the type specimens in the diagnostic characters, but they differ in having the carapace with stronger arcuate ridges on the gastric region and with two spines, instead of one spine, on the posterior branchial margin, the anterior margin of the thoracic sternite 3 being bilobed instead of nearly transverse, the thoracic sternite 4 being less elongate anteriorly, and the lateral eye-spine being stronger. Macpherson (2007) also pointed out the same difference in the shape of the thoracic sternite 3 in his topotypic material. Moreover, in one of the two Taiwanese specimens examined (ovigerous female, pcl 12.3 mm) there is a small spine laterally on each protogastric region of the carapace; this spine is not described or illustrated in the type material of *M.pycnopoda*.

As discussed by Baba (2005), *M.pycnopoda* is morphologically close to *M.lignaria* Williams & Baba, 1989 from the eastern Pacific off Oregon, but it is distinguishable from the latter species by the wider base of the rostrum, the P2–4 meri with more strongly upstanding spines on the dorsal margins, and the P2–4 dactyli being distally thicker and with straight, rather than somewhat arched, ventral margins.

## Supplementary Material

XML Treatment for Munidopsis albatrossae

XML Treatment for Munidopsis pycnopoda

## ﻿Additional information

### Author ORCIDs

Masayuki Osawa https://orcid.org/0000-0001-8600-7550

Tin-Yam Chan https://orcid.org/0000-0002-8143-0007

Chien-Hui Yang https://orcid.org/0000-0002-4594-3622

### Data availability

All of the data that support the findings of this study are available in the main text.
